# Conversion of Oximes to Carbonyl Compounds by Triscetylpyridinium Tetrakis(oxodiperoxotungsto) Phosphate (PCWP)-mediated Oxidation with Hydrogen Peroxide

**DOI:** 10.3390/molecules13061230

**Published:** 2008-06-01

**Authors:** Francesco P. Ballistreri, Ugo Chiacchio, Antonio Rescifina, Gaetano Tomaselli, Rosa M. Toscano

**Affiliations:** Dipartimento di Scienze Chimiche, Università di Catania, Viale Andrea Doria 6, Catania 95125, Italy; E-mails: uchiacchio@unict.it; gtomaselli@unict.it; rmtoscano@unict.it

**Keywords:** Oxidation of oximes, oxodiperoxotungsto complex, 1,3-dipolar cycloaddition, nitrile oxides, aldehydes.

## Abstract

Aromatic and aliphatic oximes have been deoximated in chloroform-water to the corresponding aldehydes with dilute hydrogen peroxide and triscetylpyridinium tetrakis (oxodiperoxotungsto) phosphate as catalyst. The presence of dipolarophiles in the reaction mixtures allows a competitive reaction that converts oximes into isoxazole and isoxazoline derivatives via the intermediate formation of nitrile oxide species.

## Introduction

Oximes are frequently used as carbonyl protector groups [1] from which the parent carbonyl compounds must be regenerated. Regeneration of the carbonyl compound requires the use of soft reagents that will cleave the oxime bond without modification under mild reaction conditions.

Furthermore, since oximes can also be prepared from non-carbonyl compounds, the generation of carbonyl compounds from them provides an alternative method for the preparation of aldehydes and ketones [2,3,4,5]. The traditional hydrolytic method for deprotection of oximes requires the use of strong acids and often results in low yields due to the formation of polymeric by-products, so a number of alternative methods have been developed. Some previously reported carbonyl compound deoximation methods involve oxidative or reductive protocols using, for example, pyridinium dichromate, *t*-butyl-hydroperoxide, and so on. [6,7]. Some of these reactions have different disadvantages such as long reaction times, difficulties in isolation of products and the possibility of explosions due to the presence of unstable compounds produced by strong oxidative reagents. Many oxidative deoximation methods of aldoximes cited in the literature give low yields of aldehydes due to their over-oxidation to acids.

Mo(VI) and W(VI) peroxopolyoxo complexes, whose general formula is Q_3_^+^{PO_4_[MO(O_2_)_2_]_4_}^3–^, are one of the most promising group of catalysts for the selective transfer oxygen to organic substrates [8,9]. They can be stoichiometrically used as oxidant agents or as catalysts in the oxidation processes employing dilute hydrogen peroxide. Lacunary polyoxotungstates have been also recently been screened as catalysts for H_2_O_2_oxidations under microwave irradiation [10]. Hydrogen peroxide as oxidant has the great advantage to generate only water as by-product. It has a high content of active oxygen and it is less expensive than organic peroxides and peracids. Another advantage of using these salts as oxidants comes from the possibility that the counteraction Q^+^ itself acts as a phase transfer agent when Q^+^ represents a suitable ammonium salt. In this paper we wish to report the oxidative deprotection reaction of oximes with hydrogen peroxide mediated by triscetylpyridinium tetrakis(oxodiperoxotungsto) phosphate as catalyst to yield the corresponding carbonyl compounds under mild conditions and high yields.

## Results and Discussion

Aromatic and aliphatic oximes **1** treated in water-chloroform at 30 °C with dilute hydrogen peroxide (35%, v/v) and 1 mol% of [C_5_H_5_N^+^(CH_2_)_14_CH_3_]_3_{PO_4_[WO(O_2_)_2_]_4_}^3–^ (PCWP), used as catalyst, have been transformed to carbonyl compounds **2** (Table 1).

**Table 1:** Deoximation of aldoximes **1** by oxidation with diluted hydrogen peroxide and PCWP ^a^ 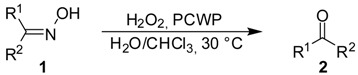

Oxime	R^1^	R^2^	Time (min.)	Conversion (%)	Yield (%)^b,c^
**1a**	C_6_H_5_	H	80	90	100
**1b**	*p*-Cl-C_6_H_4_	H	70	95	100
**1c**	C_6_H_5_	CH_3_	90	80	95
**1d**	CH(Me)(Et)	H	60	95	70
**1e**	*n*-C_7_H_15_	H	60	95	70
**1f**	–(C_5_H_10_)–		90	85	93

^a ^All the reactions have been performed in CHCl_3_/H_2_O at 30 °C employing [Oximes] = 2.5 mmol; [H_2_O_2_] = 20 mmol and [PCWP] = 0.025 mmol. ^b ^Isolated yields. ^c ^Identities of compounds have been confirmed by comparison of their MS and ^1^H-NMR spectra with those of authentic samples.

The results listed in Table 1 indicate that the reaction is successful for a variety of aliphatic and aromatic oximes. Moreover, the obtained results suggest that aldoximes are deprotected relatively faster than ketoximes.

**Scheme 1:**
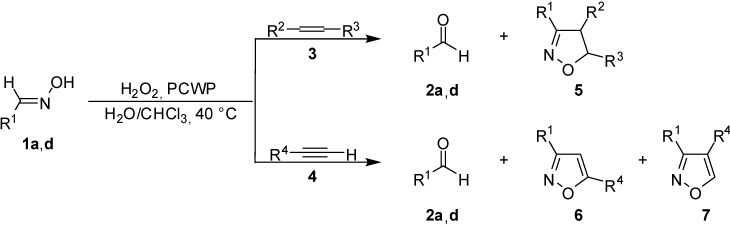


We also explored the possibility of generating nitrile oxides intermediates for the preparation of isoxazole and isoxazoline derivatives *via* 1,3-dipolar cycloaddition [11,12], to further expand the synthetic utility of the PCWP oxidation of aldoximes. *N*,*O*-Heterocycles are considered privileged structures in medicinal chemistry, as they show a wide spectrum of biological activities and have been used as antimitotic agents, antiviral compounds, antimicotics and so on [13,14]. Moreover, these compounds have several synthetically useful functionalities, masked in the rings. These functionalities can be released through ring cleavage giving easy access to a variety of open chain derivatives which are differently functionalized [15]. Thus, the reaction in chloroform-water of aldoximes **1a**,**d**, used as model compounds, and treated with dilute hydrogen peroxide (35%, v/v) and 1 mol% of [C_5_H_5_N^+^(CH_2_)_14_CH_3_]_3_{PO_4_[WO(O_2_)_2_]_4_}^3–^ (PCWP), used as catalyst, at 40 °C in the presence of alkenes **3** (1 equiv.) or alkynes **4** (2.5 equiv.), produced isoxazolines **5** or isoxazoles **6** and **7**, respectively, along with variable amounts of aldehydes **2a**,**d** (Scheme 1, Table 2).

**Table 2:** Preparation of isoxazole and isoxazoline derivatives by aldoximes oxidation in the presence of alkenes or alkynes ^a^.

Entry	R^1^	R^2^	R^3^	R^4^	Time (h)	Conv. (%)	Yields, (%)^b,c^			
							2	5	6	7
1	C_6_H_5_	H	CH_3_(CH_2_)_5_		9	84	62	17		
2	CH(Me)(Et)	H	CH_3_(CH_2_)_5_		5	89	56	6		
3	C_6_H_5_^d^	CO_2_Me	CO_2_Me		9	81	43	46		
4	CH(Me)(Et)^e^	CO_2_Me	CO_2_Me		6	100	30	30		
5	C_6_H_5_			C_6_H_5_	16	71	67		28	
6	CH(Me)(Et)			C_6_H_5_	6.5	90	49		9	
7	C_6_H_5_			CH_3_(CH_2_)_5_	6.5	83	49		32	
8	CH(Me)(Et)			CH_3_(CH_2_)_5_	3	88	33		5	
9	C_6_H_5_			CO_2_Me	8	86	30		42	13
10	CH(Me)(Et)			CO_2_Me	3	100	43		25	8

^a^All the reactions have been performed in CHCl_3_/H_2_O at 40 °C employing [Oximes] = 2.5 mmol; [H_2_O_2_] = 20 mmol and [PCWP] = 0.025 mmol. ^b ^Isolated yields. ^c ^Identities of the compounds have been obtained comparing their MS and ^1^H-NMR spectra with those of authentic samples. ^d^The reaction is stereospecific; fumarate gave *E*-adduct whereas maleate gave *Z*-adduct. ^e^The reaction has been performed with fumarate.

The results indicate that the aldehydes are the main products of the reaction, except for entries 3 and 4 (cycloadduct-aldehyde ratio 1:1) and entry 9 (cycloadduct-aldehyde ratio 1.8:1). The stereochemistry of the cycloadducts depends on the stereochemistry of the dipolarophiles (entry 3) – the *E*-adduct was obtained using fumarate and the *Z*-ones has been obtained with maleate. Moreover, all the experiments show that aromatic oximes lead to a higher yield of cycloadduct than aliphatic ones, and the presence of electron-withdrawing groups on the dipolarophile moiety increases the yield of cycloadduct (entries 3, 4, 9 and 10). The formation of isoxazole derivatives **5**–**7** supports the intermediate formation of the corresponding nitrile oxide [16]. In fact, the reaction of C_6_H_5_CH=NOH (2.5 mmol) with H_2_O_2_ (20 mmol) and PCWP (0.025 mmol) in CHCl_3_ at 40 °C, in the absence of dipolarophiles gives as main product benzaldehyde, along with a small amount of diphenylfuroxan. The same reaction followed by IR shows two significative bands: at 2250 cm^–1^ corresponding to benzonitrile oxide [17], and at 1700 cm^-1^ associated to benzaldehyde. Furthermore, the data point out that the regioselectivity of the cycloaddition process is in accordance with both steric and frontier molecular orbital interactions of the reagents [16].

**Scheme 2:**
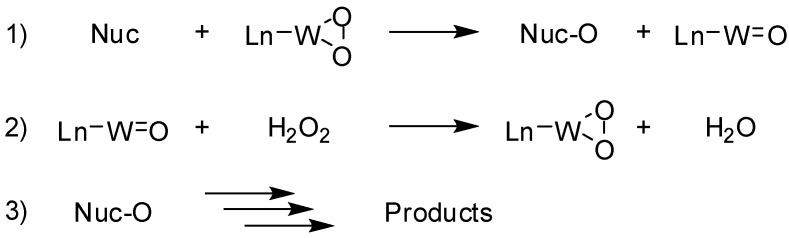


At this stage it is hard to suggest a mechanistic pathway for this reaction. It is known that peroxopolyoxocomplexes such as PCWP behave as electrophilic oxidants [18,19] and therefore the oxidation reaction might be triggered by a nucleophilic [18,20] attack of the oxime nitrogen-atom to the peroxide oxygen followed by catalytic hydrogen peroxide regeneration of the oxidant and by subsequent steps for the products formation (Scheme 2). However, it is also known that PCWP is also a good electron acceptor [21,22] and therefore the involvement of electron transfer events cannot be excluded *a priori*.

## Conclusions

In summary, aromatic and aliphatic oximes **1** can be easily deoximated in water-chloroform to the corresponding aldehydes **2 **with dilute hydrogen peroxide (35%, v/v) mediated by 1 mol% of [C_5_H_5_N^+^(CH_2_)_14_CH_3_]_3_{PO_4_[WO(O_2_)_2_]_4_}^3–^ (PCWP). The presence in the reaction mixtures of alkenes and alkynes as dipolarophiles allows a competitive reaction path which converts the oximes into isoxazole and isoxazoline derivatives through the intermediate formation of nitrile oxide species.

## Experimental

### General

Aldoximes, alkynes and alkenes (Aldrich) were distilled or crystallized before use. Chloroform (Carlo Erba, RPE) was distilled over P_4_O_10_, whereas H_2_O_2_ (35%, v/v) (Carlo Erba, RPE) was used without further purification. ^1^H- and ^13^C-NMR were obtained on a Varian Unity Inova 200 MHz spectrometer operating at 200 and 50 MHz, respectively, using CDCl_3 _as solvent and TMS as internal standard. GLC analyses were carried out on a programmable Perkin-Elmer 8420 gas chromatograph equipped with a flame ionization detector and a 25 m DB-1 capillary column. GC/MS analyses were performed on a Hewlett-Packard model 5890 gas chromatograph, using an HP-1 dimethylpolysiloxane 25 m capillary column, equipped with a Hewlett-Packard MS computerized system Model 5971A, ionization voltage 70 eV, electron multiplier 1700 V, ion source temperature 280 °C. IR spectra were recorded on a Perkin-Elmer Paragon 500 FT-IR Spectrometer using potassium bromide discs.

### Preparation of triscetylpyridinium tetrakis(diperoxotungsto)phosphate (PCWP)

To a solution of cetylpyridinium chloride (3.1 mmol) in 35% H_2_O_2_ (40 mL) has been added H3PW12O40·nH_2_O (3 g) in 35% H_2_O_2_ (10 mL), and the mixture has been stirred at 40 °C for 4–5 h. The white precipitate, after filtration, has been washed with water until all the H_2_O_2_ was removed and then dried *in vacuo* over P_4_O_10_. The IR spectrum (KBr) corresponded to the one reported in the literature [23].

### Oxidation and cycloaddition reactions: general procedure for oxidation

To a warm (30 °C) solution containing PCWP (0.025 mmol) in chloroform (5 mL) has been added a solution of aldoxime (2.5 mmol) and H_2_O_2_ (35%, 1.8 mL, 20 mmol). After the addition has been completed, the mixture has been stirred at 30 °C for an appropriate time, then the organic layer has been separated, washed with a 10% of aqueous solution of sodium bisulphite and dried over anhydrous sodium sulphate. The solvent has been removed under reduced pressure to leave a thick oil, which has been subjected to silica gel chromatography using a 30% ethyl acetate/cyclohexane mixture as eluent. The ^1^H-NMR spectra of aldehydes **2a**–**f** corresponded to the ones reported in the literature.

### General procedure for oxidation-cycloaddition

A solution of aldoxime (2.5 mmol), alkene (2.5 mmol) or alkyne (7.5 mmol) in chloroform (5 mL) and H_2_O_2_ (35%, 1.8 mL, 20 mmol) were added to a warm (40 °C) solution of PCWP (0.025 mmol) in chloroform (5 mL). After the addition was completd, the mixture was stirred at 40 °C for an appropriate time, after which the organic layer was separated, washed with a 10% aqueous solution of sodium bisulphite and finally dried over anhydrous sodium sulphate. The solvent has been removed under reduced pressure to leave a thick oil, which was subjected to silica gel chromatography using a 30% ethyl acetate/cyclohexane mixture as eluent. The ^1^H-NMR and MS spectra, for the compounds obtained in entries 1 [24], 3 [25,26], 5 [27], 7 [28], and 9 [29,30], corresponded to the ones reported in the literature.

Data for new compounds in Table 2:

*(5RS)-3-sec-Butyl-5-hexyl-4,5-dihydroisoxazole (entry 2)*: Light yellow oil; ^1^H-NMR: 0.84–0.89 (m, 6H), 1.23 (d, 3H, *J* = 6.1 Hz), 1.24–1.43 (m, 12H), 2.28 (m, 1H), 2.63 (dd, 1H, *J* = 8.4 and 16.5 Hz), 3.01 (dd, 1H, *J* = 10.2 and 16.5 Hz), 4.49 (m, 1H); ^13^C-NMR: 11.8, 14.1, 18.6, 22.7, 26.0, 26.6, 29.0, 31.7, 35.4, 35.6, 38.4, 80.6, 165.8; Anal. calcd. for C_12_H_25_NO: C, 73.88; H, 11.92; N, 6.63%. Found: C, 73.67; H, 11.94; N, 6.62%.

*Dimethyl (4RS,5RS)-3-sec-butyl-4,5-dihydroisoxazole-4,5-dicarboxylate (entry 4)*: Light yellow oil; ^1^H- NMR: 0.87 (t, 3H, *J* = 7.1 Hz), 1.15 (d, 3H, *J* = 6.1 Hz), 1.18–1.40 (m, 2H), 2.58 (m, 1H), 3.72 (s, 3H), 3.81 (s, 3H), 4.63 (d, 1H, *J* = 5.0 Hz), 5.40 (d, 1H, *J* = 5.0 Hz); ^13^C-NMR: 11.8, 18.2, 25.9, 34.8, 51.6, 52.5, 60.1, 80.8, 165.9, 167.6, 168.2; Anal. calcd. for C_11_H_17_NO_5_: C, 54.31; H, 7.04; N, 5.76%. Found: C, 54.48; H, 7.05; N, 5.75%.

*3-sec-Butyl-5-phenylisoxazole (entry 6)*: Light yellow foam; ^1^H-NMR: 0.81 (t, 3H, *J* = 7.1 Hz), 1.28 (d, 3H, *J* = 6.8 Hz), 1.38–1.69 (m, 2H), 2.87 (m, 1H), 6.31 (s, 1H), 7.30–7.78 (m, 5H); ^13^C-NMR: 12.4, 20.1, 29.3, 35.6, 102.7, 125.3, 127.8, 131.9, 132.2, 165.4, 167.3; Anal. calcd. for C_13_H_15_NO: C, 77.58; H, 7.51; N, 6.96%. Found: C, 77.35; H, 7.53; N, 6.97%.

*3-sec-Butyl-5-hexylisoxazole (entry 8)*: Light yellow oil; ^1^H-NMR: 0.88 (t, 3H, *J* = 7.1 Hz), 1.23 (d, 3H, *J* = 6.1 Hz), 1.24–1.43 (m, 10H), 1.62 (m, 2H), 2.69 (t, 2H, *J* = 6.6 Hz), 2.81 (m, 1H), 5.80 (s, 1H); ^13^C-NMR: 11.6, 14.0, 19.5, 22.4, 26.7, 27.4, 28.7, 29.2, 31.4, 33.1, 98.4, 168.3, 173.2; Anal. calcd. for C_13_H_23_NO: C, 74.59; H, 11.07; N, 6.69%. Found: C, 75.76; H, 11.04; N, 6.68%.

*Methyl 3-sec-butylisoxazole-5-carboxylate (entry 10)*: Light yellow oil; ^1^H-NMR: 0.91 (t, 3H, *J* = 7.3 Hz), 1.31 (d, 3H, *J* = 7.1 Hz), 1.44–1.78 (m, 2H), 2.95 (m, 1H), 3.96 (s, 3H), 7.03 (s, 1H); ^13^C-NMR: 12.3, 20.4, 30.1, 31.2, 53.1, 112.8, 157.6, 159.4, 171.7; Anal. calcd. for C_9_H_13_NO_3_: C, 59.00; H, 7.15; N, 7.65%. Found: C, 58.84; H, 7.18; N, 7.63%.

*Methyl 3-sec-butylisoxazole-4-carboxylate.* Light yellow oil; ^1^H-NMR: 0.93 (t, 3H, *J* = 7.3 Hz), 1.34 (d, 3H, *J* = 7.1 Hz), 1.47–1.81 (m, 2H), 2.97 (m, 1H), 3.86 (s, 3H), 9.02 (s, 1H); ^13^C-NMR: 12.8, 20.6, 29.4, 30.6, 51.1, 112.5, 154.2, 167.5, 172.1; Anal. Calcd. for C_9_H_13_NO_3_: C, 59.00; H, 7.15; N, 7.65%. Found: C, 59.11; H, 7.13; N, 7.66%.
